# Particulate matter containing environmentally persistent free radicals induces AhR-dependent cytokine and reactive oxygen species production in human bronchial epithelial cells

**DOI:** 10.1371/journal.pone.0205412

**Published:** 2018-10-11

**Authors:** Ashlyn C. Harmon, Valeria Y. Hebert, Stephania A. Cormier, Balamurugan Subramanian, James R. Reed, Wayne L. Backes, Tammy R. Dugas

**Affiliations:** 1 Department of Comparative Biomedical Sciences, Louisiana State University School of Veterinary Medicine, Skip Bertman Drive, Baton Rouge, LA United States of America; 2 Department of Pharmacology, Toxicology, and Neuroscience, LSU Health Sciences Center–Shreveport, Shreveport, LA United States of America; 3 Department of Biological Sciences, Louisiana State University, Baton Rouge, LA United States of America; 4 Department of Environmental Sciences, Louisiana State University, Baton Rouge, LA United States of America; 5 Department of Pharmacology and Experimental Therapeutics, LSU Health Sciences Center, New Orleans, LA United States of America; Utah State University, UNITED STATES

## Abstract

Particulate matter (PM) is emitted during the combustion of fuels and wastes. PM exposure exacerbates pulmonary diseases, and the mechanism may involve oxidative stress. At lower combustion temperatures such as occurs in the cool zone of a flame, aromatic compounds chemisorb to the surface of metal-oxide-containing PM, resulting in the formation of surface-stabilized environmentally persistent free radicals (EPFR). Prior studies showed that PM-containing EPFR redox cycle to produce reactive oxygen species (ROS), and after inhalation, EPFR induce pulmonary inflammation and oxidative stress. Our objective was to elucidate mechanisms linking EPFR-induced oxidant injury with increased cytokine production by pulmonary epithelial cells. We thus treated human bronchial epithelial cells with EPFR at sub-toxic doses and measured ROS and cytokine production. To assess aryl hydrocarbon receptor (AhR) activity, cells were transfected with a luciferase reporter for xenobiotic response element activation. To test whether cytokine production was dependent upon AhR activation or oxidative stress, some cells were co-treated with an antioxidant or an AhR antagonist. EPFR increased IL-6 release in an ROS and AhR- and oxidant-dependent manner. Moreover, EPFR induced an AhR activation that was dependent upon oxidant production, since antioxidant co-treatment blocked AhR activation. On the other hand, EPFR treatment increased a cellular ROS production that was at least partially attenuated by AhR knockdown using siRNA. While AhR activation was correlated with an increased expression of oxidant-producing enzymes like cytochrome P450 CYP1A1, it is possible that AhR activation is both a cause and effect of EPFR-induced ROS. Finally, lipid oxidation products also induced AhR activation. ROS-dependent AhR activation may be a mechanism for altered epithelial cell responses after EPFR exposure, potentially via formation of bioactive lipid or protein oxidation products.

## Introduction

While thermal processing of organic wastes is commonly used at Superfund sites in the U.S. and at similar locations across the world, the unintended health consequences associated with the combustion of pollutants have not yet been fully identified [1]. A major concern associated with burning hydrocarbons at these sites is the production and emission of fine (diameter <2.5 μm) and ultrafine (diameter <0.1 μm) particulate matter (PM) that may have major health impacts. Epidemiologic studies have identified a strong association between increased PM concentrations and both cardiovascular and respiratory events [2]. Increases in PM exposure correlated with the development of asthma [3–5], and exposure to fine PM (PM_2.5_) at concentrations approved by the EPA for even a short interval decreased lung function [6]. Moreover, a prospective cohort study and meta-analysis in 11 European cohorts revealed that for every 5 μg/m^3^ increase in PM_2.5_ over the course of a year, coronary events increased by 13% [7]. In a Canadian cohort, for every 10 μg/m^3^ PM_2.5_ increase, incident hypertension increased by 13%, with cardiovascular mortality increasing by 1% [8]. In addition, PM_2.5_ exposure was linked with increases in hospital admissions for cardiovascular events and chronic obstructive pulmonary disease, and on average it is estimated that PM_2.5_ exposure is responsible for approximately 200,000 deaths each year in the U.S [9]. Although numerous studies have clearly documented the health consequences of PM exposure, the underlying mechanisms driving the development of these diseases after inhalation exposure have yet to be elucidated.

Free radical formation during organic waste removal has been of concern for many years, but the radicals produced were believed to be short-lived and therefore, of limited human health impact. However, our colleagues [10] and numerous other laboratories worldwide [11–16] have identified that PM resulting from the thermal processing of hazardous organic wastes contain environmentally persistent free radicals (EPFR). The presence of these free radicals has been documented in ambient air PM collected from cities across the U.S., and persist for days to months [17,18]. Formation of EPFR occurs when incompletely combusted hydrocarbons chemisorb to metal-containing PM during thermal processing [19,20]. This causes a reduction of the metal and the formation of an organic free radical that is stabilized by the surface of the metal-containing PM, allowing it to persist in the environment for days [21]. This pollutant-particle system has unique characteristics beyond that of their organic or metal constituents and can participate in extended redox cycling in the air, soil or biological fluids [22,23], leading to its production of large quantities of reactive oxygen species (ROS), including superoxide and hydroxyl radical. This production of ROS suggests that the risks associated with airborne PM may be underestimated. Considering that a quarter of the U.S. population lives within a 4 mile radius of a Superfund site [24], with approximately 30% of these sites remediated using thermal processes [25], and that significant levels of EPFR-containing PM have been detected near these sites [26], studies examining the biological mechanisms of toxicity of these particles and their exposure risks are imperative.

The ability of EPFR to redox cycle is likely one of the most biologically important characteristics of the particle system that may contribute to accelerating cardiopulmonary disease pathogenesis. Prior EPFR exposure studies demonstrated the development of oxidative stress both *in vitro* and *in vivo* [22,27–28]. Cell culture studies using human bronchial epithelial cells (BEAS-2B) reported increased ROS in cells after exposure, as well as increases in inflammation and cell death [28]. Exposure of rodents to EPFR increased ROS levels in the lungs, heart and blood and reduced pulmonary and cardiac function [29–30].

Many inhaled components of PM have been shown to induce oxidative stress, increase inflammatory cytokine secretion and activate the aryl hydrocarbon receptor (AhR) system in the lung [31]. While EPFR-containing PM was shown to increase both oxidative stress and inflammation [30], their effect on activation of the AhR is unknown. While it is generally known that the AhR mediates the toxic effects of certain hydrocarbons, more recently, the AhR was shown to regulate normal physiologic processes, as well [32–33]. Prior studies demonstrated that exogenous ligands that activate the AhR are typically planar, hydrophobic halogenated or polycyclic aromatic hydrocarbons [34]. However, other experiments aimed at identifying endogenous ligands for the receptor showed that prostaglandin metabolites of oxidative stress at high levels were capable of activating AhR [35–36]. Moreover, the AhR and its biomarkers are highly expressed in the lung [31], and its activation leads to pulmonary inflammation [31]. Therefore, the objective of this study was to test whether oxidative stress and the pro-inflammatory response induced by EPFR treatment of pulmonary epithelial cells involves AhR activation and if so, to elucidate the mechanisms and mediators involved.

## Materials and methods

### Generation of combustion-derived EPFR

To study the cytotoxic effects of EPFR, we utilized the laboratory-generated combustion particles we used in prior *in vitro* and *in vivo* studies [28,36]. These EPFR were synthesized through the heating of 1, 2-dichlorobenzene (DCB) or 2-monochlorophenol (MCP), together with CuO as a source of metal and amorphous SiO_2_ particles at 230°C, as previously described [20,37]. These EPFR particle systems were denoted as DCB230 and MCP230, indicating the organic component and the temperature at which they were formed. Note that Si and copper are common components of PM [38], dichlorobenzene is a pollutant frequently found in air and included on the Hazardous Substances List [39,40], and chlorinated phenols are common contaminants in Superfund sites, particularly those of former wood treatment facilities [41]. Moreover, both DCB230 and MCP230 exhibit an electron paramagnetic resonance (EPR) spectrum indicative of an oxygen centered phenoxyl and/or *o*-semiquinone radical [20]. These particles thus exhibit very similar radical signatures by EPR compared to urban-collected EPFR [42] but offer many advantages, including lack of contaminants and size uniformity. Our group reported that both types of EPFR produce large amounts of hydroxyl radical in solution, in biological fluids and in cultures of pulmonary epithelial cells [22]. Transmission electron microscopy showed that these EPFR exhibit a particle size of ~100–200 nm when suspended in aqueous solution [27]. As controls, some experiments compared 5% CuO-fumed silica, denoted as SiO_2_/CuO [22], that do not contain EPFR *per se* but may produce low levels of ROS due to Fenton chemistry associated with a transition metal [22]. In a few experiments, controls also included size-identical amorphous silica (SiO_2_) or particles containing MCP physisorbed to SiO_2_, but containing no transition metal (MCP50), synthesized as described previously [22].

### Culture and treatment of cells

Human bronchial epithelial cells (BEAS-2Bs; ATCC) were cultured at 37°C with 5% CO_2_ in RPMI medium supplemented with 5% fetal bovine serum (FBS; v/v), 25 mM sodium bicarbonate, 0.5% glutamine and 1% antibiotic/anti-mycotic solution. Cells were trypsinized, transferred to 25 cm^2^ flask and grown to confluence prior to particle exposure. In addition to MCP230 and DCB230, cells were also exposed to the size-identical non-EFPR controls described above. α-tocopherol (α-T; 100 μM) or 3,4-dimethoxyflavone (3, 4-DMF; 10 μM) were added to particle suspensions in some experiments to reduce oxidative stress or to block the aryl hydrocarbon receptor, respectively. Cells were exposed to each particle treatment at a concentration of 50 or 100 μg/cm^2^. In prior studies, these concentrations were shown to be moderately toxic to pulmonary epithelial cells [22], with little cell death occurring until 24h of treatment, facilitating studies of the EPFR mechanisms of action. Particles were weighed and to ensure adequate suspension, were added to a solution of BEGM (Bronchial Epithelial Cell Growth Medium, ATCC) containing no phenol red and lacking serum but with 0.02% Tween 80, a non-ionic surfactant used in food products, in cosmetics and to stabilize aqueous formulations of medications for parenteral use. Each solution was then pulsed using a 100W Microson XL2000 Ultrasonic Cell Disruptor (10 s on, 10 s off) for 2 minutes at 10% power to ensure the particles were completely suspended prior to adding the particle solution to the cells. Exposures were for 2, 4, 6, 8 or 24 hours depending on the analysis performed.

### Cellular ROS production

BEAS-2B cells were exposed to particles with and without α-T (α-tocopherol) or 3,4-DMF (3,4-dimethoxyflavone) for 2–24 hours at a concentration of 50 μg/cm^2^. Intracellular ROS production was measured using the fluorescent probe dihydrorhodamine 123 (DHR; 5 μM), which was preloaded on BEAS-2B 45 minutes prior to particle exposure. Next, cells were washed with PBS and exposed to the particles for another 40 minutes. After this exposure, BEAS-2B cells were washed twice with PBS and a final volume of 100 μL of PBS was added to each of the wells before measuring fluorescence at Ex/Em = 485/528 nm.

### Aryl hydrocarbon receptor activation

BEAS-2B cells were transfected with an inducible XRE-responsive firefly luciferase construct and a constitutively expressing Renilla luciferase vector (Qiagen) prior to particle exposures. Briefly, BEAS-2Bs were grown to ~80% confluence then transfected with XRE-Lipofectamine (Invitrogen) complexes 48 hours. Cells were washed and then exposed to either vehicle, MCP230, DCB230 or CuO/SiO_2_, each with and without α-T or 3,4-DMF. As a positive control for AhR activation, some cell samples were exposed to 10 nM of the AhR agonist 2,3,7,8-tetrachlorodibenzodioxin (TCDD). Assessment of AhR activation by lipid metabolites was carried out using this same protocol. In some experiments, transfected BEAS-2B cells were exposed to lipid metabolites known to be associated with oxidative stress and oxidant producing enzymes, including PGF_2α_, 8-iso-PGF_2α_, thromboxane (TXB2), metabolites of thromboxane, and the lipoxygenase product LXA_4_, alongside a TCDD positive control. The Dual Luciferase Assay (Invitrogen) was performed after an 8 hour exposure and activation of AhR was calculated as a ratio of XRE-driven luciferase:renilla luminescence. To normalize the data to account for differences in responses between passages, data were expressed as a percent of the positive control TCDD.

### Cytokine production

Cytokine production was assessed using enzyme-linked immunosorbent assays (ELISAs), performed using conditioned medium from BEAS-2B cells exposed for 8 hours to particles. IL-6 was measured in cells exposed to particles at a concentration of 50 μg/cm^2^. Because initial studies demonstrated lower levels of TNF-α compared to IL-6, TNF-α was measured in medium from BEAS-2B cells exposed to concentrations of 50 and 100 μg/cm^2^ MCP230 and DCB230 for 8 hours. IL-6 and TNF-α assays were performed using 100 μL of medium in commercially available ELISA kits (eBiosciences). All assays were carried out in accordance with manufacturer’s suggested protocols. Cytokine levels in pg/mL were then normalized to cell protein, determined using the BCA assay, and were expressed as pg/mg.

### Inhibition of particle uptake

To test whether AhR activation was dependent upon cellular uptake of the particles, 48h after transfection with XRE reporter, cells were treated with a mix of 10 μM wortmannin (phosphoinositide 3-kinase inhibitor)/ 10 μg/mL chlorpromazine (inhibits clathrin-mediated endocytosis)/ 2.5 μg/mL filipin III (inhibits caveolae-mediated uptake) to inhibit all known cell uptake pathways. In some experiments, cells were exposed to 80 μM dynasore (to inhibit dynamin in the regulation of endocytosis) or 10 μg/mL cytochalasin D (to inhibit receptor-mediated and fluid-phase endocytosis). BEAS-2Bs were then exposed to vehicle, MCP230 or DCB230 at a concentration of 50 μg/cm^2^ for 8 hours, and activation of the AhR was expressed as a ratio of luciferase:renilla luminescence. Note that 8h was a time point where in prior studies, cells remained viable and where in pilot studies [22], AhR activation was at its highest level.

### Knockdown of AhR and ROS production

To determine the role of AhR activation in intracellular ROS production, AhR was knocked down in cells using AhR siRNA (Dharmacon, Lafayette, CO), with scrambled siRNA used as a control (Dharmacon). The efficiency of knockdown was confirmed using western blot analysis for AhR protein levels. BEAS-2B cells were exposed to vehicle or MCP230 for 8 hours at a concentration of 50 μg/cm^2^. Cells were then washed with PBS, preloaded with 5 μM DHR for 45 minutes, washed again with PBS and then exposed to the particles for another 40 minutes. The particle-containing media were then aspirated and the cells were washed twice with PBS. Finally, 100 μL of PBS was added to the cells and fluorescence was measured at Ex/Em = 485/528 nm.

### Quantitative PCR for assessing *Cyp1a1* mRNA expression

Real time PCR (qRT-PCR) was utilized to determine *Cyp1a1* expression levels in cells exposed to MCP230 versus cells exposed to control particles and vehicle only. After an 8-hour exposure, media containing the particles was removed and the cells were washed with PBS, trypsinized and then collected into microcentrifuge tubes. Cells were pelleted *via* centrifugation, washed again with PBS and stored frozen at –80°C until RNA was isolated. Total RNA was extracted using the RNeasy Protect Mini Kit (Qiagen) and the isolation procedure was performed according to the manufacturer provided instructions. cDNA was synthesized from 1 μg of RNA using the High-Capacity RNA to cDNA Kit (Thermo Fisher). qRT-PCR was performed using RT2 Sybr Green Master Mix (Qiagen) and a 7900HT Real-Time PCR 96 well plate system (Applied Biosystems). *Cyp1a1* and *HPRT1* (Life Sciences) primers were used. Levels of *Cyp1a1* mRNA were calculated using the mathematical formula 2^−ΔΔCt^ recommended by Applied Biosystems.

### Statistics

For experiments assessing endpoints at a single time point, one-way ANOVA was utilized, with significant differences between individual data points elucidated using Holm-Sidak’s multiple comparison tests. In cases where both treatment and time were compared, two-way ANOVA was used. Data in figures are expressed as means ± SD. In all cases, p<0.05 was accepted as significant. All statistical analyses were conducted using GraphPad Prism version 6.05 (La Jolla, CA) software.

## Results

### EPFR exposure increases ROS production in BEAS-2B cells

**Fig 1:**
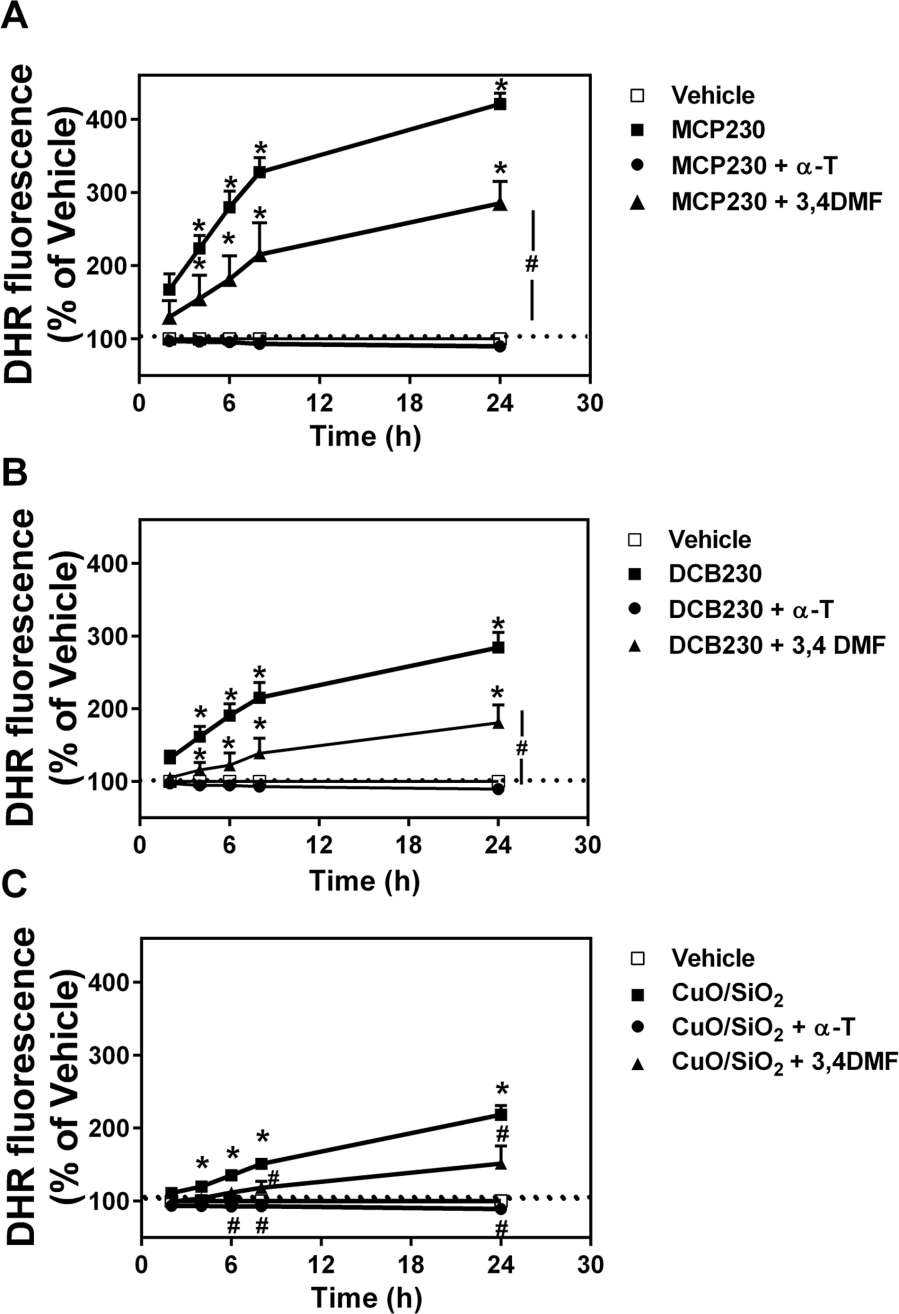
Reactive oxygen species (ROS) production is increased in epithelial cells treated with EPFR. BEAS-2Bs were exposed for 0-24h to 50 μg/cm^2^ EPFR (MCP230 or DCB230; A and B, respectively), compared to CuO/SiO_2_ (C). ROS were assessed by incubation of cells with the fluorescent probe dihydrorhodamine (DHR). In some experiments, cells were co-treated with α-T, an antioxidant, or 3,4-dimethoxyflavone (3,4-DMF), an aryl hydrocarbon receptor (AhR) antagonist. Data were expressed as a % of vehicle. Data are means ± SD for n = 3–5 per treatment group. *Denotes p<0.05 compared to controls. ^#^Indicates *p*<0.05 compared to the particle treatment group (i.e., MCP230, DCB230 or CuO/SiO_2_).

Compared to vehicle, BEAS-2Bs treated with MCP230 or DCB230 exhibited significantly increased ROS production at each of the five time points over 2–24 hours, with MCP230 increasing levels by ~ 163%– 421% and DCB230, by 132–284%. (Fig 1A and 1B, respectively). However, these increased ROS levels were significantly reduced when cells were co-incubated with the antioxidant α-T, with levels not elevated above that of the vehicle (Fig 1A and 1B). A reduction in ROS production was also noted when cells were co-incubated with the AhR antagonist 3,4-DMF. Specifically, in the presence of 3,4-DMF, MCP230 increased ROS levels by only 130–285% over 2–24 hours, and DCB230 increased levels by only 105–180% of vehicle (Fig 1A and 1B). While not as dramatic an increase as that observed for EPFR exposure, BEAS-2Bs exposed to CuO/SiO particles also increased ROS production; this could be inhibited by both α-T and 3, 4-DMF pre-treatments (Fig 1C).

### EPFR increase aryl hydrocarbon receptor activation

**Fig 2:**
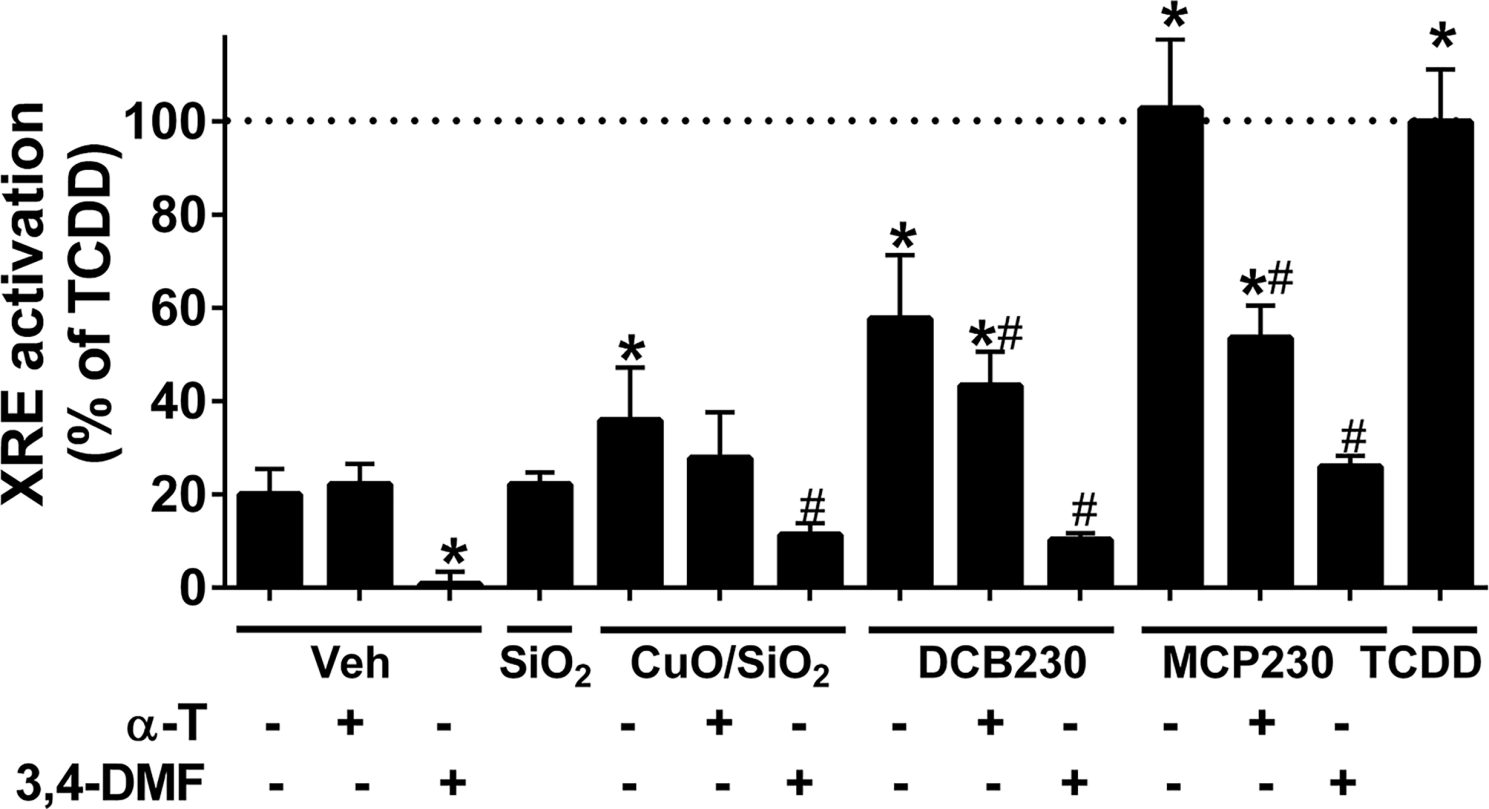
EPFR induce AhR activation. BEAS-2Bs were transfected with an XRE reporter for AhR activation and then exposed to 50 μg/cm^2^ non-EPFR (SiO_2_), CuO/SiO_2_, non-EPFR but capable of producing low levels of oxidants, compared to the EPFR MCP230 and DCB230 for 8h. Cells were then lysed and a dual luciferase assay was performed. The AhR agonist TCDD (25 nM) served as a positive control. In some experiments, cells were co-treated with 100 μM of the antioxidant α-T or 10 μM of the AhR antagonist 3,4-DMF. To normalize for differences between cell passages, data are expressed as % of TCDD. Data are means ± SD for n = 3–10. *Denotes *p*<0.05 compared to controls. ^#^Indicates *p*<0.05 compared to the particle treatment group (i.e., MCP230, DCB230 or CuO/SiO_2_).

Luciferase reporter assays for XRE were used to determine AhR activation after exposure of cells to EPFR. As explained above, data were expressed as a percent of the positive control TCDD, so as to normalize for differences in responses between cell passages. First, control particles containing no radicals, i.e., amorphous SiO_2_, exhibited no increase in XRE activation compared to vehicle (Fig 2), and treatment with the antioxidant α-T alone also exhibited no effect. On the other hand, compared to vehicle, treatment with the AhR antagonist 3,4-DMF significantly decreased levels of XRE activation (Fig 2). Exposure to DCB230 and MCP230 increased XRE activation ~3- and 5-fold, respectively, while treatment with CuO/SiO_2_ increased XRE activation by 64%. Co-treatment with AhR antagonist dramatically inhibited XRE activation observed for the EPFR–MCP230 and DCB230 –as well as for CuO/SiO_2_. Finally, co-treatment with antioxidant significantly reduced XRE activation observed for MCP230 and DCB230, but did not significantly attenuate that observed for CuO/SiO_2._

### IL-6 and TNF-α are increased in BEAS-2B cells exposed to EPFR

**Fig 3:**
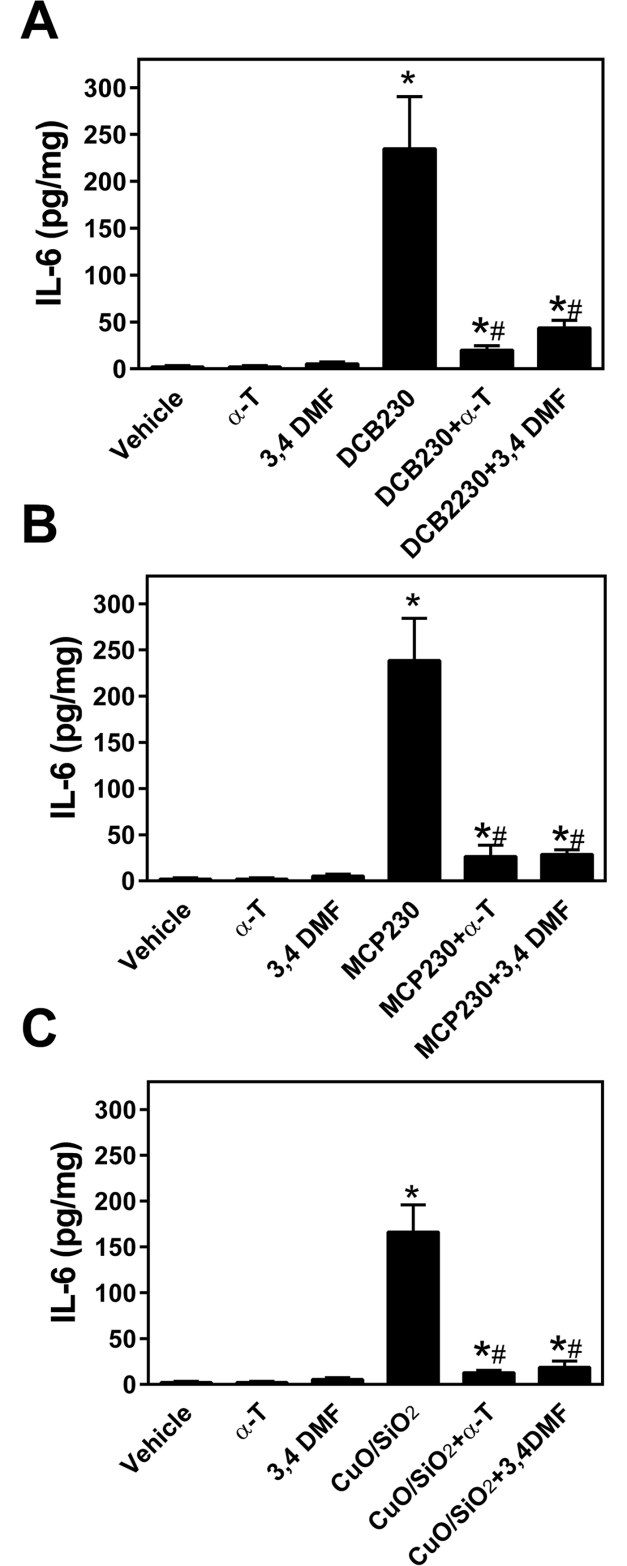
EPFR stimulate IL-6 production. BEAS-2Bs were exposed to 50 μg/cm^2^ of EPFR (DCB230 or MCP230; **A** and **B**, respectively) compared to size-identical CuO/SiO_2_ (**C**) for 8h, and IL-6 production in the culture supernatants was measured by ELISA. Some cells samples were co-treated with the antioxidant α-T or the AhR antagonist 3,4-DMF. Results were normalized to cell protein. Data are means ± SD for n = 3–5. *Denotes *p*<0.05 compared to vehicle. ^#^Indicates *p*<0.05 compared to the particle treatment group (i.e., MCP230, DCB230 or CuO/SiO_2_). TNF-α production was likewise increased after exposure to EPFR, although larger concentrations of EPFR (e.g., 100 μg/cm^2^) were required for observing elevations comparable to that observed for IL-6 (Fig 4).

Compared to vehicle and control groups, EPFR significantly increased the production of IL-6, with levels increasing from 2.2 ± 0.5 to 240 ± 16 and 230 ± 20 pg/mg protein for MCP230 and DCB230, respectively (Fig 3A and 3B). However, these levels were dramatically attenuated by the addition of antioxidant (i.e., α-T) or AhR antagonist (3,4-DMF; Fig 3A and 3B). IL-6 levels were also measured after exposure to CuO/SiO and found to be elevated in the BEAS-2B medium, although not to the extent noted after exposure to EPFR, with levels elevated to only 170 ± 11 pg/mg (Fig 3C). CuO/SiO-induced IL-6 production was also reduced by the addition of α-T and 3,4-DMF (Fig 3C).

**Fig 4:**
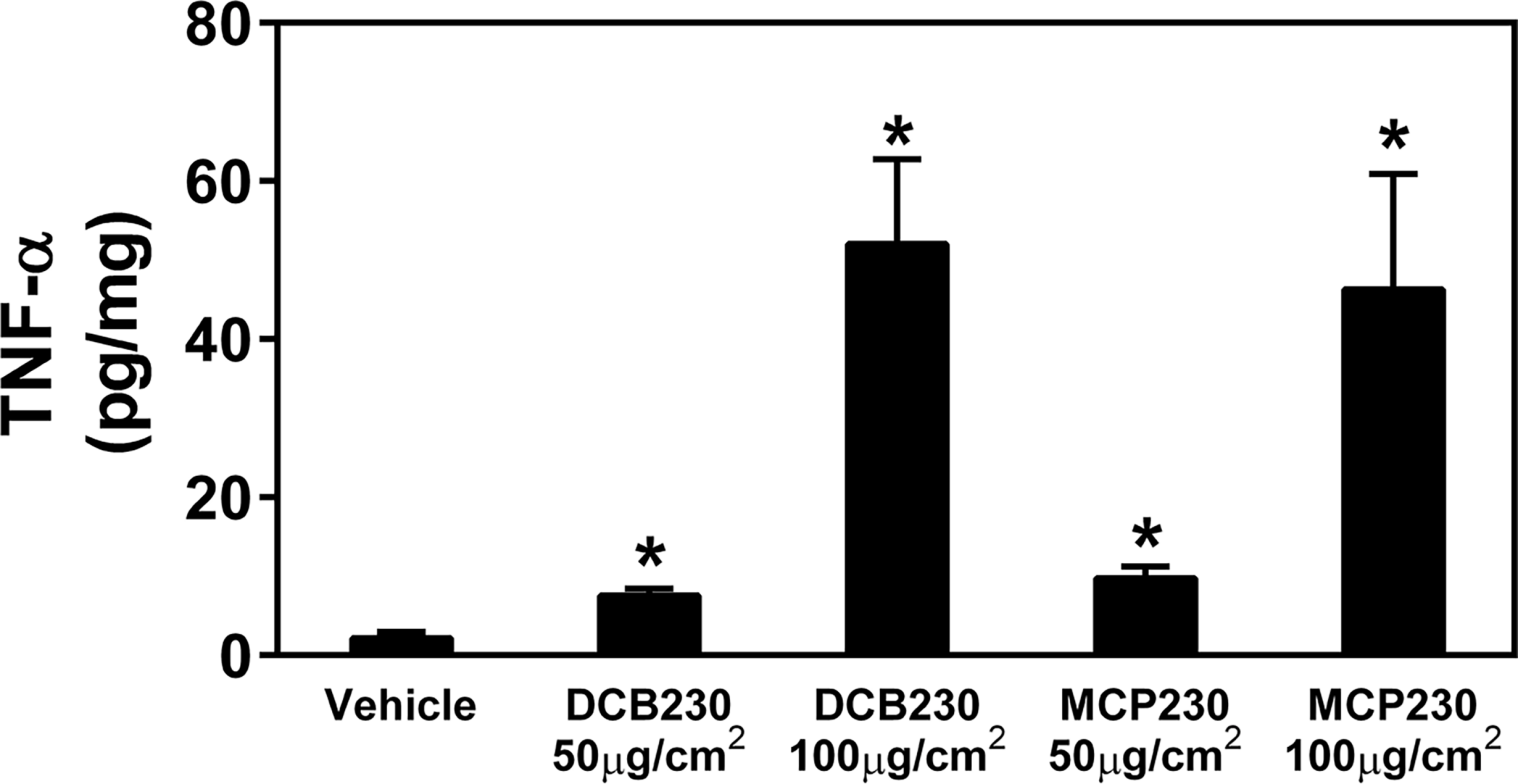
EPFR increase TNF-α release. BEAS-2Bs were exposed to EPFR (DCB230 or MCP230) at 50 μg/cm^2^ or 100 μg/cm^2^ for 24h, and TNF-α production in tissue culture supernatants was measured. Results were normalized to cell protein. Data are means ± SD for n = 3–5. *Denotes *p*<0.05.

### AhR activation is not dependent on particle uptake

**Fig 5:**
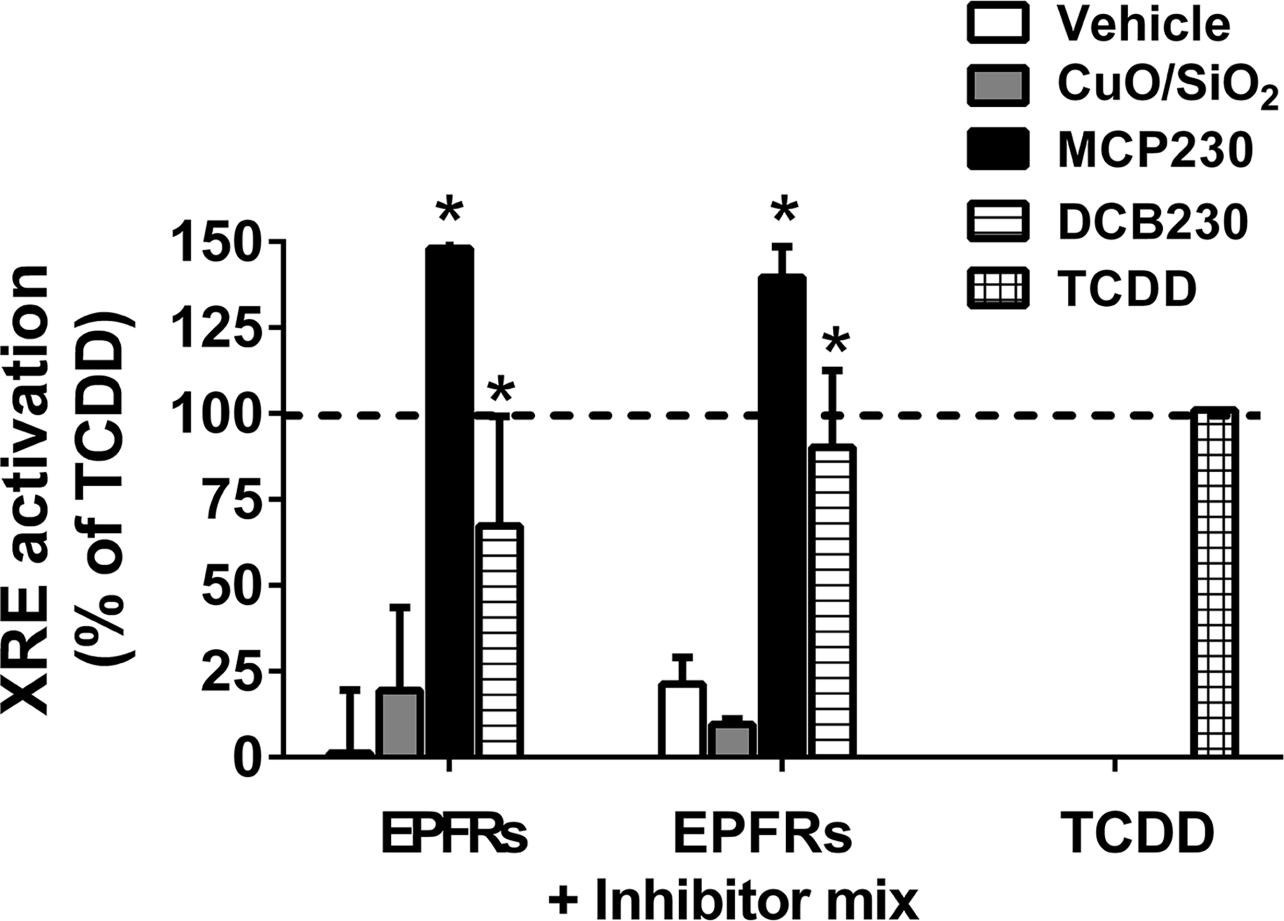
EPFR-induced AhR activation is not altered by cellular uptake blockers. BEAS-2Bs were treated with 50 μg/cm^2^ MCP230, DCB230 or CuO/SiO_2_, including inhibitors of clathrin-independent endocytosis (wortmannin), clathrin-dependent endocytosis (chlorpromazine), and caveolae-mediated uptake (Fillipin III) applied as a mixture. Inhibitors cytochlasin D and dynasore produced similar findings (not shown). Data are means ± SD for n = 3–4. *p<0.05 vs. control.

To test whether the particles were acting directly to increase the activation of the AhR, a mixture of uptake inhibitors (wortmannin/chlorpromazine/filipin III) were administered to the cells along with each of the particle groups and the activation of AhR, determined. Again, in this experiment, the activation of the receptor was expressed as a percent of the response observed for the positive control TCDD. Exposure to MCP230 and DCB230 increased AhR activation to 210 ± 5.4 and 160 ± 8.9%, respectively, but this level of activation was not altered by the addition of the uptake inhibitor mixtures (Fig 5). Activation of the receptor was also measured after exposure to CuO/SiO, both with and without the uptake inhibitors, and again, no difference noted in activation in the presence of the inhibitors (Fig 5). Moreover, individual treatment with dynasore (to inhibit dynamin in the regulation of endocytosis) or cytochalasin D (to inhibit receptor-mediated and fluid-phase endocytosis) produced no alteration in XRE activation after any of the EPFR treatments–MCP230, DCB230 or CuO/SiO_2_ (not shown).

### ROS production is partially decreased after knockdown of the AhR gene

**Fig 6:**
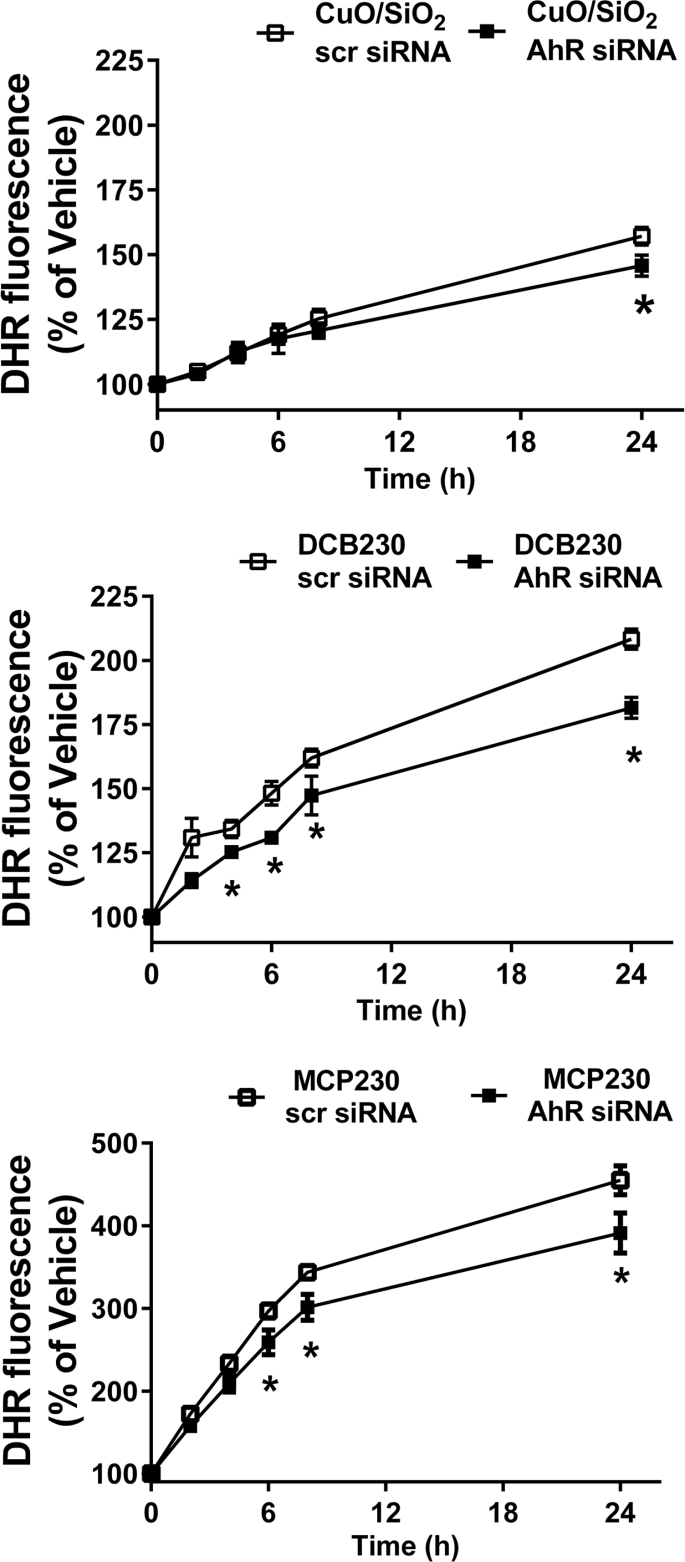
siRNA against AhR only minimally blocks levels of ROS. BEAS-2B were treated with siRNA against AhR or scrambled siRNA, and cells were exposed to 50 μg/cm^2^ MCP230, DCB230 or CuO/SiO_2_ for 4, 6, 8 and 24h. Increases in ROS levels were determined as DHR fluorescence. Data are means ± SD for n = 3 experiments. *Denotes *p*<0.05 compared to vehicle.

To test whether ROS production was elevated in response to AhR activation, siRNA was used to knockdown AhR expression. Western blot analysis revealed that compared to cells treated with scrambled siRNA, AhR siRNA reduced AhR protein levels by ~50% (not shown). Similar to levels noted in the initial ROS experiment, BEAS-2B cells treated with scrambled siRNA and then dosed with MCP230 demonstrated ROS levels ranging from 170 ± 2.1–460 ± 10.0% of vehicle over the 2–24 hour time course. While only slightly diminished (*p*<0.05), cells exhibiting reduced AhR expression using siRNA demonstrated ROS production ranging from 160 ± 3.0–390 ± 14% (Fig 6).

### Quantitative PCR shows increased expression of *Cyp1a1* after exposure to EPFR

**Fig 7:**
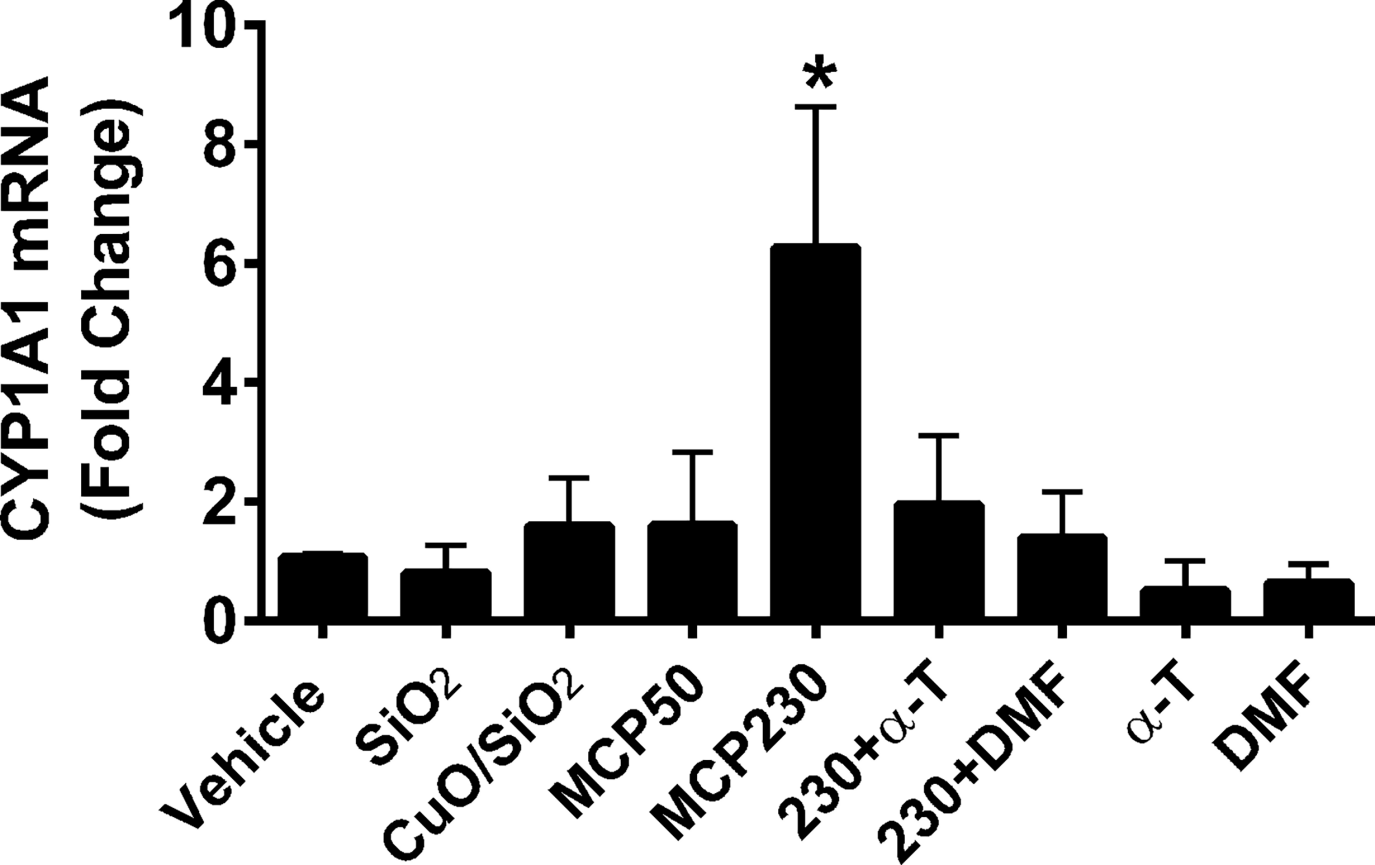
*Cyp1a1* mRNA is upregulated in BEAS-2Bs treated with EPFR in an AhR and oxidative stress-dependent manner. BEAS-2B cells were exposed to 50 μg/cm^2^ of particles for 8 h. mRNA levels of CYP1A1 relative to the housekeeping gene HPRT using qPCR, and data were expressed as a fold change compared to vehicle. Data are means ± SD for n = 3 experiments. *Denotes *p* <0.05.

*Cyp1a1* mRNA levels were significantly increased 6.2-fold in cells exposed to MCP230 compared to cells treated with vehicle only (Fig 7). mRNA levels in cells exposed to control particles (SiO_2_, CuO/SiO_2_, MCP50) and in those co-treated with MCP230 + α-T and MCP230+3,4-DMF were not significantly altered compared to that of the vehicle. In addition, treatment with α-T or 3,4-DMF only did not alter baseline levels of *Cyp1a1* mRNA.

### 2, 3-dinor-TXB_2_ and LXA_4_ increase aryl hydrocarbon receptor activation

**Fig 8:**
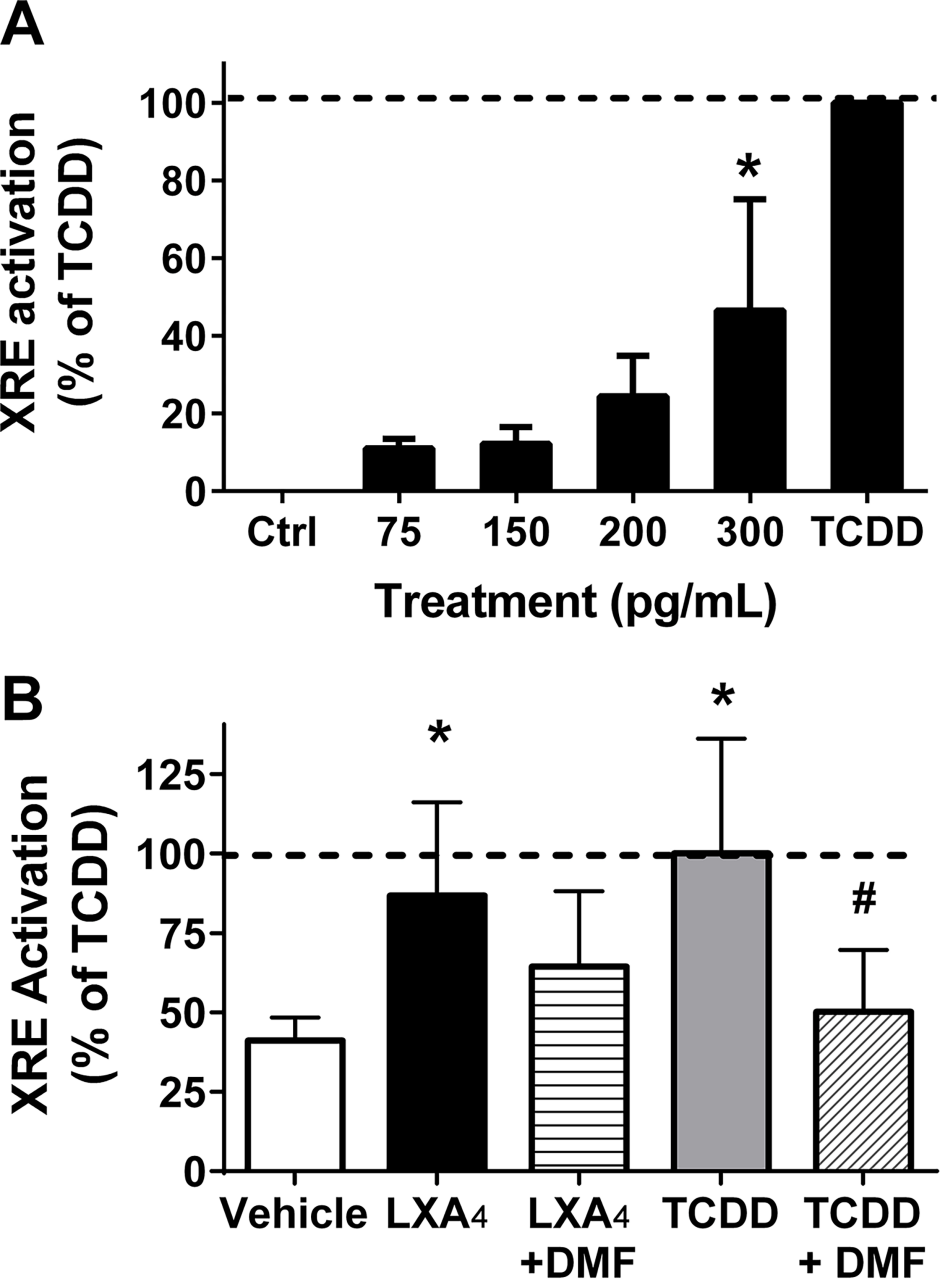
Lipid metabolites promote AhR activation. Epithelial cells were treated with physiologic concentrations of 2,3 dinor-TXB2 (A) or 1.8 ng/mL LXA_4_ (B) compared to 10 nM TCDD, as a positive control. AhR inhibitor DMF was used in some experiments. AhR activation was assessed using a luciferase reporter for XRE activation. Results expressed as % of TCDD (10 nM). Data are means ± SD for n = 3–4. *p<0.05 vs. control. #Denotes *p*<0.05 compared to the experimental treatment group (i.e., LXA_4_ or TCDD).

To test the hypothesis that small molecule intermediates act to mediate EPFR-induced AhR activation, BEAS-2B cells transfected with the XRE reporter. The cells were then exposed to increasing physiologic concentrations of a number of commercially available lipid metabolites known to be associated with oxidative stress and oxidant producing enzymes, including PGF_2α_, 8-iso-PGF_2α_, thromboxane (TXB2), metabolites of thromboxane, and the lipoxygenase product LXA_4_ for 8 h, a time point where maximal AhR activation had been observed for EPFR. Results were expressed as percent of maximum AhR activation, which was determined using 10 nM of the positive control TCDD. Of the metabolites tested, only 2,3-dinor TXB2 and LXA_4_ exhibited significant XRE activation. Specifically, when 2,3-dinor-TXB_2_ was administered in four separate concentrations: 75 pg/mL, 150 pg/mL, 200 pg/mL and 300 pg/mL, percent activation increased in a dose-dependent manner and was statistically significant at the 300 pg/mL concentration, where XRE activation was 47 ± 17% of the response observed for TCDD (Fig 8A). Similar to what was reported by Chiaro et al. [43– 44], LXA_4_ also significantly increased AhR activation at very low levels, i.e., to 87 ± 15% of TCDD at 1.8 ng/mL LXA_4_. To demonstrate the dependence of this result on AhR activation, in this experiment, we included co-treatment with AhR antagonist 3,4-DMF. In this case, XRE activations levels were reduced nearly to the level observed for treatment with vehicle only (Fig 8B).

## Discussion

The AhR is a ligand-activated transcription factor that is highly expressed in barrier sites such as the lung. Its pronounced expression in the lung epithelium coincides with a high expression of AhR biomarkers CYP1A1 and CYP1B1 [45]. The AhR serves to regulate epithelial barrier function, preventing inspired luminal components from entry into the sub-epithelial layer. Moreover, the epithelium serves as a barrier to modulate lung responses to environmental and infectious stimuli [46], and barrier disruption can promote lung inflammation and disease pathogenesis through their production of chemokines/cytokines and ROS [46]. AhR activation in these lung epithelia has been shown important in modulating the production of mucins, in support of their barrier function, and of ROS, to protect against invading pathogens [47]. Moreover, pulmonary epithelial AhR activation can induce cytokine production to modulate the lung’s immune response. As an example, *via* AhR activation, diesel exhaust particles have been shown to stimulate cytokine release from bronchial epithelial cells [48]. Moreover, while airborne particulate matter was shown to induce IL-6 expression in BEAS-2B *via* NFkB [49], in these same cells, AhR activation, through a pathway involving NFkB and RelA, promoted IL-6 expression [50].

Using BEAS-2B cells, we sought to understand the molecular mechanisms by which EPFR-containing particulate matter promoted epithelial cytotoxicity and inflammatory responses. We utilized a unique EPFR particle system where we can control for the composition of the particulate. For example, we can expose cells to particles containing an EPFR, i.e., with both an organic and a redox active metal, or we can synthesize a particulate containing only metal or one with only the organic. Using this unique model system, we showed that EPFR are strong activators of the AhR (Fig 2), and that this activation in turn stimulates cytokine production (Figs 3 and 4). Because cytokine production could be blocked using both an AhR antagonist and an antioxidant, we rationalized that EPFR-mediated AhR activation and ROS production may have causal roles in the observed increase in cytokine production. However, given known structural requirements for AhR activation by an organic [51], neither dichlorobenzene nor monochlorophenol would be expected to induce AhR activation. Moreover, since AhR activation did not require cellular uptake (Fig 5), it seemed unlikely that the particle itself served as a ligand to induce its activation.

A potential mechanism began to emerge, in which EPFR induced cytokine production was a concomitant of either an AhR-induced ROS production, or an ROS-induced AhR activation. Given the weight of evidence prior to our studies–reports that AhR activation leads to ROS production *via* increased CYP1A expression [52–53]—one would presume that ROS were a result of AhR activation rather than a cause. However, we showed in our previous studies that EPFR themselves are large source of ROS, as they redox cycle to produce ROS independent of their interaction with a biological system [22]. Thus, to test whether EPFR-derived ROS were a cause of AhR activation, we measured AhR activation using both an XRE luciferase reporter and *Cyp1a1* expression in cells treated with and without an antioxidant or an AhR antagonist. To our surprise, AhR activation was dramatically attenuated by both treatments (Figs 2 and 7). Note that these results are in agreement with our prior report indicating that EPFR induce *Cyp1a1* expression in an AhR-dependent manner in dendritic cells, and that AhR activation in A549 cells is inhibitable by antioxidant co-treatment [54]. However, in other studies where we blocked AhR using siRNA and measured ROS, ROS levels were attenuated by at most 10% even with a 50% knockdown of AhR levels (Fig 6). Therefore, with EPFR containing particles, AhR activation is a source of only a fraction of the total ROS produced. With EPFR exposure, ROS are likely both a cause and an effect of AhR activation.

**Fig 9:**
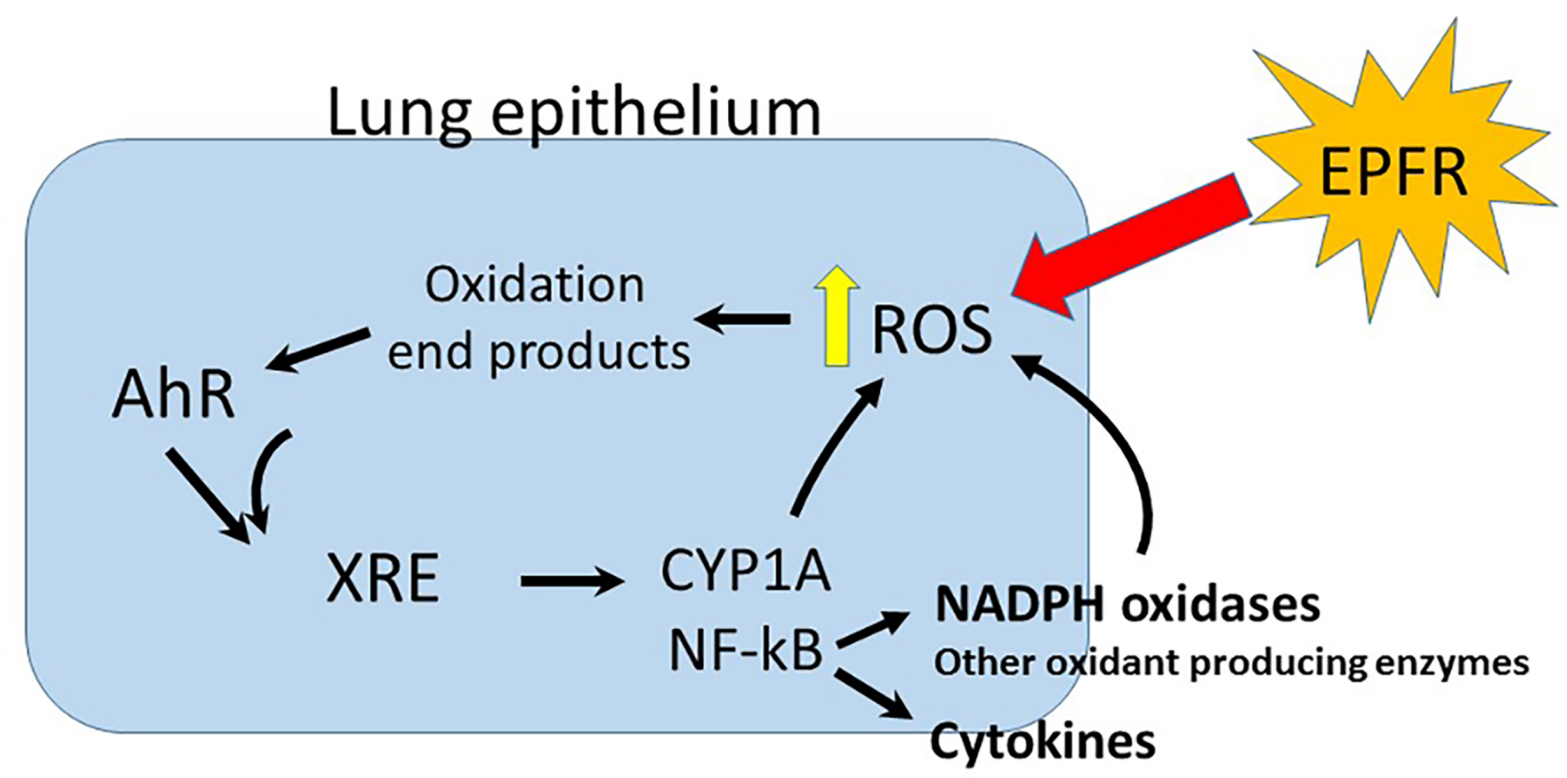
Proposed mechanism of AhR activation by EPFR.

Our next objective was to probe the link between EPFR-induced ROS and AhR activation. As AhR is a nuclear receptor, we first tested whether EPFR required internalization prior to AhR activation. In studies utilizing cocktails of drugs known to inhibit the various pathways for endocytosis, we showed that uptake of the EPFR was not required (Fig 5). These findings suggested to us that perhaps the EPFR induce oxidation of the lipid membrane and/or its membrane proteins, and products of this oxidation in turn promote AhR activation within the cell. These oxidation products then serve as ligands for the AhR, in turn promoting its activation. In turn, this AhR activation increases the production of cytokines and the expression of ROS-producing enzyme systems such as CYP1A, and perhaps even other oxidant producing enzymes (see Fig 9). In support of this hypothesis, lipid [43–44] and tryptophan [55] metabolites have recently been identified as putative endogenous ligands for the AhR. Numerous oxidation products of lipids and proteins are known, albeit not yet tested for their AhR activating properties. To demonstrate the feasibility that in our model system, a lipid oxidation product could potentially induce AhR activation, we tested a number of lipid metabolites that are commercially available in our XRE luciferase reporter assay in BEAS-2Bs. We identified two lipid metabolites exhibiting strong AhR activation in our cells–LXA_4_, as reported by Chiaro et al [43,44], as well as 2,3-dinor-TXB2 (Fig 8). 2,3-dinor TXB2 is a beta oxidation product of TXB2 often measured in urine as a marker for cyclooxygenase-mediated metabolism of lipids *in vivo* [56]. To our knowledge, this is the first demonstration of 2,3-dinor TXB2 as an AhR activator. Although we have no knowledge of whether either of both of these metabolites, or potentially other lipid metabolites not yet identified, have causal roles in EPFR-induced AhR activation, our findings demonstrate the plausibility of mechanism involving lipid intermediates.

## Conclusions

From the compilation of studies conducted here, we propose a mechanism wherein EPFR induce ROS production and oxidative stress, culminating in the oxidation of biomolecules to yield small molecule metabolites capable of activating the AhR. This AhR activation then increases CYP1A1 expression, further stimulating cellular ROS production. It is likely, though, that expression of other ROS producing enzymes are likewise enhanced. Given the association of AhR activation and NFkB signaling [50], and the role of NFkB signaling in regulating the expression of the NADPH oxidase [57] and other oxidant-producing enzymes, it is possible that additional sources of EPFR-induced oxidant production are yet to be elucidated. In any event, given our findings for AhR activation by lipid oxidation products and the plausibility that these or other protein oxidation products may mediate EPFR-induced AhR activation, lipidomic and/or proteomic analyses for identifying these intermediates appear in order. Moreover, given the widespread expression of AhR in tissues distal to the lung and the potential for systemic circulation of these metabolites, their elucidation could prove useful for linking mechanisms for cardiovascular or other diseases known associated with PM inhalation exposures.
